# Impact of maternal characteristics on human milk oligosaccharide composition over the first 4 months of lactation in a cohort of healthy European mothers

**DOI:** 10.1038/s41598-019-48337-4

**Published:** 2019-08-13

**Authors:** Tinu Mary Samuel, Aristea Binia, Carlos Antonio de Castro, Sagar K. Thakkar, Claude Billeaud, Massimo Agosti, Isam Al-Jashi, Maria Jose Costeira, Giovanna Marchini, Cecilia Martínez-Costa, Jean-Charles Picaud, Tom Stiris, Silvia-Maria Stoicescu, Mireille Vanpeé, Magnus Domellöf, Sean Austin, Norbert Sprenger

**Affiliations:** 1 Nestlé Research, Société des Produits Nestlé S.A., Lausanne, Switzerland; 2 Nestlé Research, Société des Produits Nestlé S.A., Singapore, Singapore; 3 Hôpital des enfants, CHU Pellegrin, Bordeaux, France; 4 Ospedale del Ponte, Varese, Italy; 5 Al Jashi Isam Private Med. Practice, Bucharest, Romania; 6 Instituto de Investigação em Ciências da Vida e Saúde, Braga, Portugal; 7 Karolinska University Hospital, Stockholm, Sweden; 8 Hospital Clínico Universitario, University of Valencia, Valencia, Spain; 9 Hôpital de la Croix Rousse, Lyon, France; 10 Oslo University Hospital, Oslo, Norway; 11 Polizu Hospital, Bucharest, Romania; 12 Umeå University, Umeå, Sweden

**Keywords:** Nutrition, Medical research

## Abstract

Human milk oligosaccharide (HMO) composition varies among lactating mothers and changes during the course of lactation period. Interindividual variation is largely driven by fucosyltransferase (FUT2 and FUT3) polymorphisms resulting in 4 distinct milk groups. Little is known regarding whether maternal physiological status contributes to HMO variability. We characterized the trajectories of 20 major HMOs and explored whether maternal pre-pregnancy body mass index (ppBMI), mode of delivery, or parity may affect milk HMO composition. Using longitudinal breastmilk samples from healthy mothers (n = 290) across 7 European countries, we characterized HMO composion and employed mixed linear models to explore associations of maternal characteristics with individual HMOs. We observed HMO-specific temporal trajectories and milk group dependencies. We observed relatively small but significant differences in HMO concentrations based on maternal ppBMI, mode of delivery and parity. Our findings suggest that HMO composition to be regulated time-dependently by an enzyme as well as substrate availability and that ppBMI, mode of delivery, and parity may influence maternal physiology to affect glycosylation marginally within the initital period of lactation. Our observational study is the largest European standardized and longitudinal (up to 4 months) milk collection study assessing HMO concentrations and basic maternal characteristics. Time of lactation and milk groups had the biggest impact on HMO variation. Future studies need to elucidate these observations and assess the physiological significance for the breastfed infant.

## Introduction

Human milk (HM) is the optimal source of nutrition for infants. It contains numerous complex lipids, proteins, carbohydrates, micronutrients and bioactives that change dynamically both over a single feed and throughout the course of lactation. Likely, this reflects the changing nutritional requirements of the infant^1,2^. One of the most dominant HM components are the human milk oligosaccharides (HMOs) with estimated concentrations in mature HM of about 5–15 g/L^3,4^. Structurally a wide variety of HMOs are formed by a range of glycosyltransferases. These elongate lactose by the addition of lacto-N-biose or N-acetyllactosamine units through β1-3 or β 1-6 linkages to produce a variety of core structures. Both lactose and the core structures can be fucosylated through α1-2, α1-3 or α1-4 linkages and/or sialylated through α2-3 or α2-6 linkages.

While HMOs are well known for their role as a substrate in stimulating the growth of beneficial bacteria in the gastrointestinal tract of infants^5–7^, emerging research data suggest that they may exert effects through multiple mechanisms in a structure function specific way. These include (i) anti-infective properties by reducing pathogen attachment and subsequent infection, (ii) strengthening the gut barrier function and (iii) immunomodulation^8–12^. Findings from studies across different population groups have consistently shown a time-dependent reduction of several HMOs during the course of lactation^13–18^ and that the synthesized oligosaccharides vary among different lactating women^13,19^.

Among the factors explaining part of the inter-individual HMO variability are the genetic variations affecting the activity of fucosyltransferase 2 and 3 (FUT2 and FUT3) enzymes that are encoded by the Secretor and Lewis gene respectively^4,16,20–22^. Based on the *FUT2* and *FUT3* polymorphisms, 4 distinct milk groups can be defined. Besides genetics, other maternal and environmental factors may at least partly influence HMO composition, and thereby possibly impact infant and child health outcomes. As of today, there is limited data available from only two studies to suggest a correlation between HMOs and maternal factors, such as body weight, body mass index (BMI), parity and age^23,24^.

In the context of a multicenter European observational study to characterize the HM composition in the first four months of lactation (Atlas of Human Milk Nutrients study)^25^, we set out to characterize the HMO trajectories by FUT2/FUT3-dependent milk groups and to understand whether, and to what extent, maternal factors such as pre-pregnancy BMI (ppBMI), parity, and mode of delivery may explain part of the HMO compositional variability. Our study aims to provide new impetus in this area of limited knowledge and to add to the existing body of evidence on the temporal changes in HMOs during the first four months of lactation.

## Results

### Description of study population

**Figure 1:**
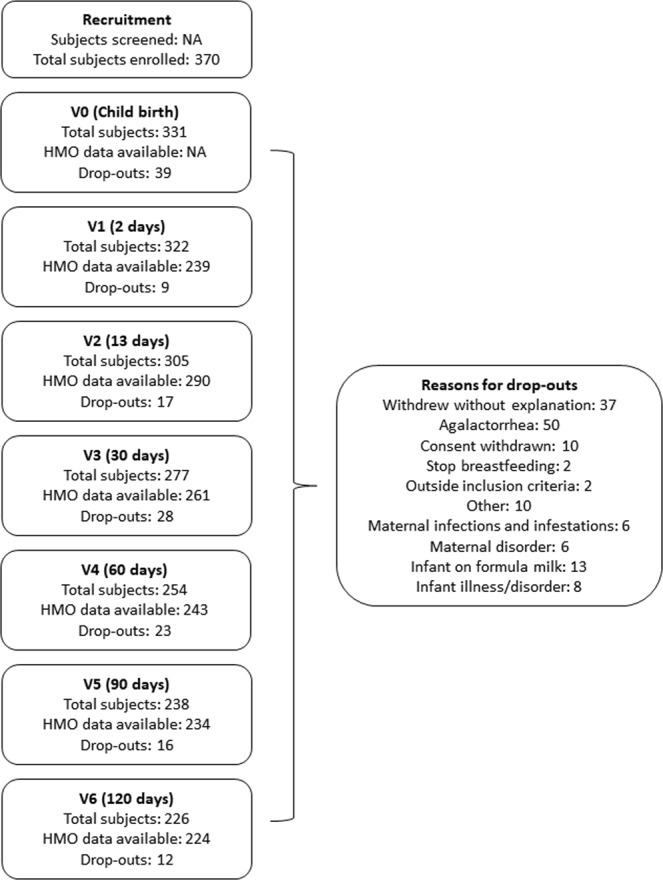
Study design and flow. *Detailed information in the Supplementary Information.

In total, 370 women were enrolled from seven European countries, Spain (ESP), France (FRA), Italy (ITA), Norway (NOR), Portugal (PRT), Romania (ROU) and Sweden (SWE) based on the eligibility criteria (Fig. 1, Table 1). 226 mothers completed the study. HMO data were available for N = 239, 290, 261, 243, 234, 224 individuals at 2 (0–3) d, 17 ± 3 d, 30 ± 3 d, 60 ± 5 d, 90 ± 5 d, and 120 ± 5 d (visits V1-V6), respectively. The main reasons for drop-outs were: Without explanation (37 women), agalactorrhea (50 women) and other reasons such as feeding infant with infant formula for more than 7 consecutive days, deciding to withdraw from the study or stopping breastfeeding to return to work (57 women) (Fig. 1, Supplementary Table 1).

**Table 1:** Demographic and maternal characteristics.

		Total number of participants	Milk group 1	Milk group 2	Milk group 3	Milk group 4	*P*-value
Proxies	**FUT2-FUT3**		POS-POS	NEG-POS	POS-NEG	NEG-NEG	
		290	209 (72.0)	51 (17.6)	20 (6.9)	10 (3.5)	
Country n (%)	**Spain**	9	5 (55.6)	4 (44.4)	0 (0.0)	0 (0.0)	0.005
	**France**	83	65 (78.3)	9 (10.8)	7 (8.4)	2 (2.4)	
	**Italy**	13	6 (46.2)	4 (30.8)	2 (15.4)	1 (7.7)	
	**Norway**	10	9 (90.0)	1 (10.0)	0 (0.0)	0 (0.0)	
	**Portugal**	95	54 (56.8)	28 (29.5)	9 (9.5)	4 (4.2)	
	**Romania**	40	35 (87.5)	2 (5.0)	1 (2.5)	2 (5.0)	
	**Sweden**	40	35 (87.5)	3 (7.5)	1 (2.5)	1 (2.5)	
Infant sex n (%)	**F**	130 (44.8)	99 (47.4)	19 (37.3)	9 (45.0)	3 (30.0)	0.454
	**M**	160 (55.2)	110 (52.6)	32 (62.7)	11 (55.0)	7 (70.0)	
Mean prepregancy weight in kg (SD)		61.68 (7.73)	61.64 (7.59)	61.43 (8.49)	62.27 (7.00)	62.80 (9.21)	0.944
Mean prepregnancy height in cm (SD)		164.88 (6.00)	165.31 (5.83)	163.42 (5.91)	162.62 (7.14)	167.97 (5.71)	0.022
Mean prepregnancy BMI (SD)		22.69 (2.61)	22.55 (2.50)	23.01 (3.00)	23.59 (2.61)	22.20 (2.63)	0.254
Prepregnancy BMI (%)	**Normal**	229 (79.0)	172 (82.3)	37 (72.5)	13 (65.0)	7 (70.0)	0.139
	**Overweight**	61 (21.0)	37 (17.7)	14 (27.5)	7 (35.0)	3 (30.0)	
Mean weight loss postpartum in kg (SD)		10.11 (4.55)	10.10 (4.81)	10.34 (3.76)	9.43 (4.51)	10.68 (3.63)	0.895
Mean age (SD)		31.20 (4.16)	30.98 (4.04)	32.08 (4.39)	31.10 (4.66)	31.50 (4.48)	0.405
Mode of delivery n (%)	**C-section**	72 (24.8)	54 (25.8)	10 (19.6)	6 (30.0)	2 (20.0)	0.736
	**Vaginal**	218 (75.2)	155 (74.2)	41 (80.4)	14 (70.0)	8 (80.0)	
Parity n (%)	**1**	215 (74.1)	158 (75.6)	32 (62.7)	16 (80.0)	9 (90.0)	0.632
	>**1**		51 (24.4)	19 (37.3)	4 (20.0)	1 (10.0)	
	**2**	58 (20.0)	38 (18.2)	15 (29.4)	4 (20.0)	1 (10.0)	
	**3**	16 (5.5)	12 (5.7)	4 (7.8)	0 (0.0)	0 (0.0)	
	**5**	1 (0.3)	1 (0.5)	0 (0.0)	0 (0.0)	0 (0.0)	
Mean gestational age, weeks (SD)		39.38 (1.19)	39.35 (1.17)	39.25 (1.23)	39.90 (1.25)	39.60 (1.17)	0.181

Maternal characteristics are summarized in Table 1. Mean age at recruitment was 31.2 years (standard deviation, SD: 4.1). 44.8% of mothers had a female infant, 74.1% gave birth the first time and 26.8% gave birth by Cesarean section (C-section). Mean gestational age was 39.4 weeks (SD: 1.2). Mean pre-pregnancy maternal weight was 61.7 kg (SD: 7.7) and based on ppBMI, 229 women were normal-weight (79%) and 61 women were overweight (21%).

The baseline clinical and anthropometric data are shown by all mothers and by milk group (Supplementary Fig. 1). No significant differences were found among the 4 milk groups, except for maternal height (*p* = 0.022) and subject distribution by country (*p* = 0.005). 72% of samples were assigned to milk group 1 (FUT2^+^-FUT3^+^), 17.6% to milk group 2 (FUT2^−^-FUT3^+^), 6.9% to milk group 3 (FUT2^+^-FUT3^−^) and 3.5% to milk group 4 (FUT2^–^-FUT3^−^) (Table 1, Supplementary Fig. 2). The distribution of milk groups was not the same in all counties; France (n = 83), Sweden (n = 40), Romania (n = 40) and Norway (n = 10) have similar distributions, with milk group 1 representing between 76–90% of the population and milk group 2 representing 5–13% of the population. Portugal (n = 95), Italy (n = 13) and Spain (n = 9) seem to have a different milk group distribution, with milk group 1 representing 50–57% of the population (Table 1, Supplementary Fig. 2).

### HMO trajectories through the first 4 months of lactation

**Figure 2:**
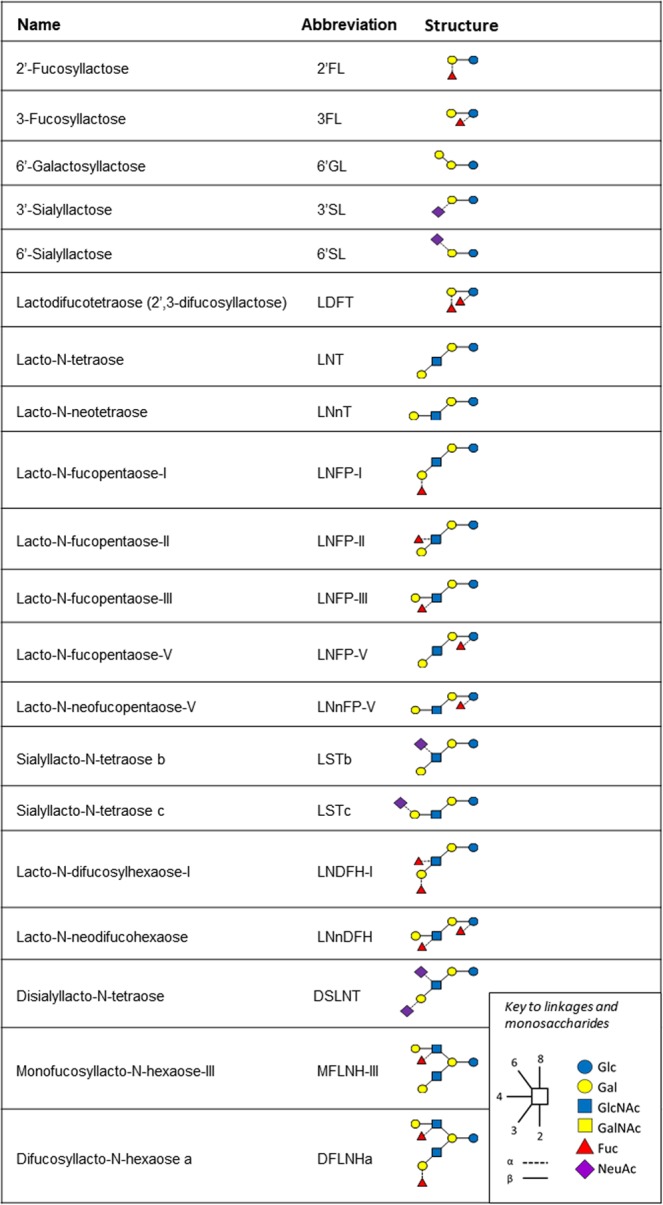
The name and structure of the analysed oligosaccharides. Monosaccharides composing the oligosaccharides: Glc, glucose; Gal, galactose; GlcNAc, N-acetyl-glucosamine; GalNAc, N-acetyl-galactosamine; NeuAc, N-acetyl-neuraminic acid.

We quantified the 20 major HMOs, as listed and illustrated in Fig. 2. Total amount of HMOs, estimated by summing the mean of the individual quantified HMOs changes over time of lactation from day 2 to day 120 with a mean (SD) of 13138 mg/L (775 mg/L) and 6650 mg/L (383 mg/L), respectively (p < 0.01; q < 0.01). Total amount of HMOs is different between milk groups 1, 2 and 4, as well as 2 versus 3 or 4 and 3 versus 4 (all p < 0.01, q < 0.01), but not between milk group 1 and 3.

**Figure 3:**
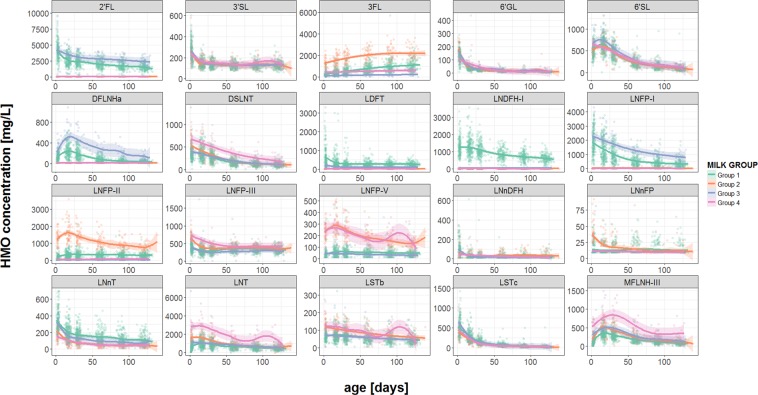
Trajectories of HMO concentrations during the first 4 months of lactation separated by milk group. The solid lines represent the smoothing curves via local polynomial regression (LOESS – Locally Weighted Scatter-plot Smoother) and the shaded area represents the 95% confidence interval. (Details on statistical differences between milk groups can be found in Supplementary Table 6).

**Figure 4:**
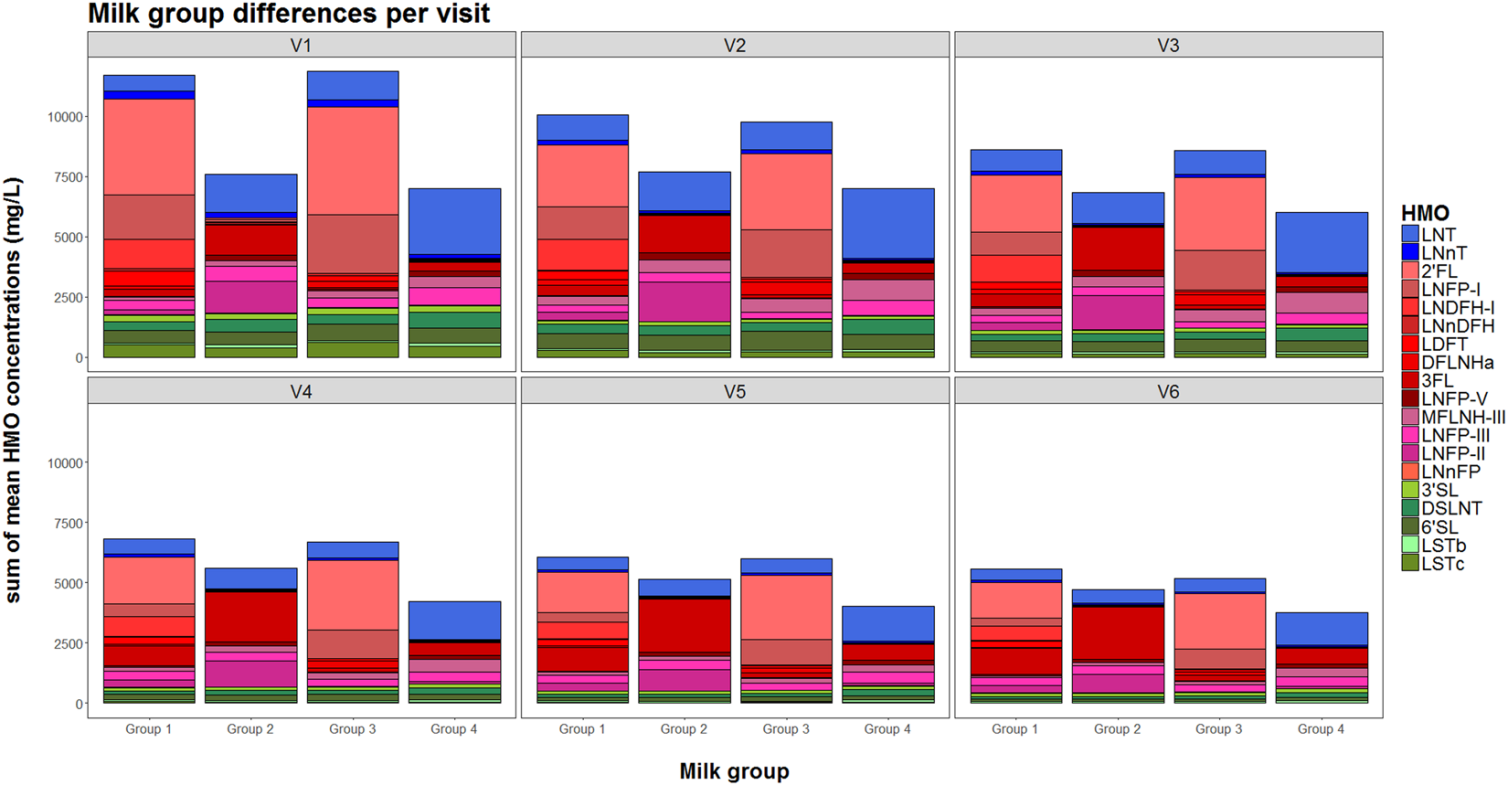
HMO profile distribution by milk group and visit as sum of the quantified HMOs.

As illustrated in Figs 3 and 4, for most HMOs the concentration is higher at early stages of lactation and decreases later (for all from day 2 to day 120, p < 0.01; q < 0.01). The most noticeable exception is 3FL, which increases from day 2 to day 120 from a mean (SD) concentration of 421 mg/L (452 mg/L) to 1209 mg/L (717 mg/L) (p < 0.01; q < 0.01).

### HMO differences by milk groups

We categorized all mothers in one of four milk groups that are characterized by the presence and absence of specific α-1,2 and α-1,4- fucosylated HMOs (Supplementary Fig. 1). All 20 quantified HMOs are plotted over time and by milk group (Figs 3 and 4).

2′FL, LNFP-I and DFLNHa are all at their highest concentration in milk group 3 (FUT2^+^-FUT3^−^) and second highest in milk group 1 (FUT2^+^-FUT3^+^) (for all p < 0.01; q < 0.01). LNnT is highest in milk groups 1 and 3 (p < 0.05; q < 0.05). LNDFH-I and LDFT are at their highest concentrations in milk group 1, and are present at very low concentrations (or below quantification limit) in other milk groups (for all p < 0.01; q < 0.01). 3FL, LNFP-II and LNnFP-V are at their highest concentrations in milk group 2 (FUT2^−^-FUT3^+^) (for all p < 0.01; q < 0.01), while MFLNH-III, DSLNT, LNFP-III and LNT are at their highest concentrations in milk group 4 (FUT2^−^-FUT3^−^) (for all p < 0.01; q < 0.01). LNFP-V and LSTb are at their highest concentrations in milk groups 2 and 4 (for all p < 0.01; q < 0.01). Milk group categories seem not to affect the concentrations of 3′SL, or LNnDFH and only slightly affect 6′SL, LSTc, or 6′GL.

### Effects of maternal factors on HMOs

#### Pre-pregnancy BMI (ppBMI)

We analyzed the effect of ppBMI by comparing the concentrations of HMOs between overweight (OW) and normal weight (NW) women at six different time points over the first four months of lactation, adjusted for milk group.

OW women had significantly higher concentrations of 3′SL, 6′GL and DSLNT (at day 2), 6′SL (at day 17) and LNFP-V (at day 90 and 120), while lower concentrations of LNnT (at day 2), LNT (at day 30 and 90) and LNFP-V (at day 60) compared to NW women (p < 0.05 for all). Upon correction for multiple testing only 3′SL and 6′GL remained significantly different (Supplementary Table 3). The magnitude of the effect observed were generally low, and for 3′SL and 6′GL 22 and 29 mg/l, respectively.

#### Mode of delivery

**Figure 5:**
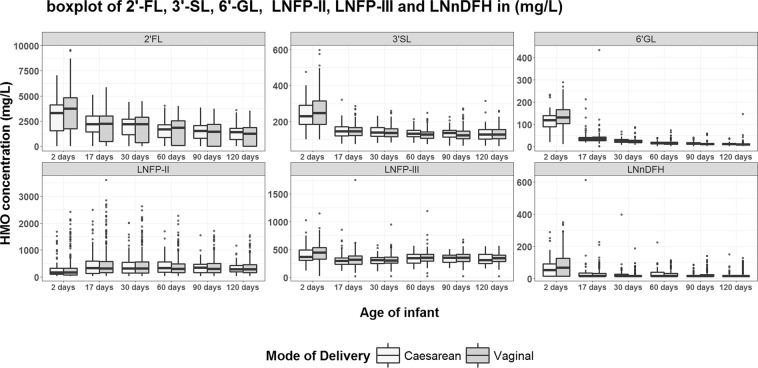
HMO concentration over time by mode of delivery.

We explored the effect of delivery mode on the HMO concentrations over the first 4 months of lactation, adjusted for milk group, ppBMI and parity (Fig. 5).

Women giving birth through C-section (CS) had significantly higher concentrations of LNT (at day 2) and 6′SL (at day 30) (p < 0.05 for both), and significantly lower concentrations of 2′FL, 3′SL, 6′GL, LNFP III, LNnDFH and LNFP II (at day 2) (p < 0.05 for all) compared to those giving birth by vaginal delivery (VB). Upon correction for multiple testing 3′SL, 2′FL and 6′GL remained significantly different (Supplementary Table 4). There were no significant differences in the concentrations of any of the other HMO at any other time point between women delivering through CS or VB.

#### Parity

We analyzed the effect of parity on HMO composition by comparing HMO concentrations in HM between primiparous (PP) and multiparous (MP) women over the first 4 months of lactation, adjusted for milk group and ppBMI.

PP women had significantly higher concentrations of DSLNT (at day 2) and LNnT (at day 17) (p < 0.05 for both), and significantly lower concentrations of 6′GL and 3FL (at day 2) and LNFP II and LNFP V (at day 17) (p < 0.05 for all) compared to MP women. Upon correction for multiple testing no HMO remained significantly different (Supplementary Table 5). None of the other HMO concentrations significantly differed between these two groups and there were no significant differences at any other time points.

## Discussion

Milk group defined by the presence or absence of functional FUT2 and FUT3 enzymes is known to have a significant influence on the HMO profile^16,21,26^. We used primarily 2′FL, and LNFP-II concentrations as proxies for FUT2 and FUT3 activities, respectively. Our data are in good agreement with those of Thurl *et al*.^16,21^ and Gabrielli *et al*.^26^, who observed the same presence/absence of the different fucosylated HMOs in the different milk groups as observed in our study. Categorizing HM samples into milk groups explains a large part of the interindividual HMO variability and needs to be considered together with time of lactation when exploring other factors that may explain HMO variability.

In different European countries approximately 77% of the population is expected to be phenotypically FUT2 (Secretor) positive and about 88% of Caucasians are expected FUT3 (Lewis) positive^27,28^. On average, 77.2% (SD 15.3) and 91.4% (SD 8.2) of our HM samples across Europe were phenotypically FUT2 and FUT3 positve, respectively. Because we had a relatively low number of participants in some of our sites a country by country comparison is not necessarily meaningful. Yet, comparison between some countries with relatively high numbers of participants like Portugal (n = 95) and France (n = 83) indicates a different distribution of milk groups. Others, like Sweden (n = 40) and Romania (n = 38) seem to have an almost identical distribution, and more similar to France. The three remaining countries (Italy, Norway and Spain) have far too few subjects to yield a meaningful conclusion about milk group distribution. A similar variation in milk group distribution between countries was also observed in a previous study^23^ that covered a broader geographical region including sites in North America, South America, Africa and Europe. In that study, however, milk samples were only categorized in 2 milk groups defined by a functional FUT2 enzyme (equivalent to the combination of milk groups 1 and 3 versus the combination of milk groups 2 and 4).

Categorizing HM samples helps not only to understand HMO variability across geographies and individuals, but also highlights HMO interdependencies and regulation based on enzyme as well as donor- and acceptor-substrate availabilities. For the dominant HMOs 2′FL and LNFP-I characteristic of milk groups 1 and 3 we see similar trajectories with the highest concentrations observed at the start of lactation followed by a gradual decrease, and with concentrations higher in group 3 than group 1. This suggests that there may be competition between FUT2 and FUT3 enzymes for the GDP-fucose substrate, because lower concentrations of oligosaccharides containing α-1,2-linked fucose residues are seen in milk group 1. Gabrielli *et al*.^26^ observed a similar profile, except that the LNFP-I concentration for milk group 3 peaked at day 10 of lactation rather than at the earlier timepoint. In our study, DFLNHa (α-1,2 and α-1,3-fucosylated) also had similar behaviour to 2′FL and LNFP-I but with the peak concentration appearing around day 13, similar to MFLNH III, the α-1,3-monofucosylated version DFLNHa. This together with the observed trajectories for 2′FL and LNFP-I seems to indicate that FUT2 activity is particularly high very early in lactation and that during lactation there is increasing competition for substrates by other upcoming fucosyltransferase activities like FUT3.

Consistent with the above observation, LNFP-II and LNFP-V both have their peak also around day 13, which for LNFP-II is in agreement with previous data^26^. 3FL, like LNFP-II, is present at highest concentrations in milk group 2 samples. However, unlike LNFP-II, and rather unique among the measured HMOs, the 3FL trajectory shows increasing concentration from early to later stages of lactation with presence in all 4 milk groups. Seemingly, lactose is still available in later stages as acceptor substrate for fucosylation by FUT3 unlike LNT and LNnT. LNFP-V, like 3FL, contains an α-1,3-linked fucose residue, however, unlike 3FL, its concentration appears to be independent of the activity of FUT3 since the concentrations are similar in both milk group 2 and 4. This implies that FUT3 is not responsible for α-1,3-fucosylation of the reducing end glucose on LNT. To the best of our knowledge, this is the first time such an observation has been made.

LNT is the core structure for many fucosylated HMOs (e.g. LNFP-I, -II, -V, LNDFH-I). Our results imply that there is a limited availability of LNT, and when FUT2 is active, it out-competes the other enzymes for access to this core structure. Indeed, when both FUT2 and FUT3 are inactive (milk group 4) we also see that LNT is at its highest concentration, as observed before^26^. Similarly, we observe that DSLNT and LSTb, which are non-fucosylated but sialylated LNT based HMOs, are higher in milk group 2 and 4, respectively, compared to the other milk groups. This indicates that sialylation competes with fucosylation for acceptor substrates like LNT. However, sialylation of lactose and LNnT resulting in the HMOs 3′SL, 6′SL, and LSTc seems less affected by milk groups suggesting acceptor substrate specific regulatory mechanisms and activities.

Although a large part of HMO variability is explained by the phenotypically determined milk groups, an additional important part might be explained by the difference between homo- and heterozygotes for the functional FUT2 and FUT3 alleles. In our cohort, we do not have genetic data to evaluate this aspect.

On the other hand, maternal physiological status, such as ppBMI, and birth conditions, such as mode of delivery and parity, might also affect maternal glycosylation and thus HMO composition. A recent cross-sectional study with 410 healthy breastfeeding mothers from 11 international cohorts indicates correlations between maternal weight, BMI and several HMOs including 2′FL and other fucosylated HMOs^23^. However, in that study an inverse correlation was reported between maternal weight, BMI and DSLNT, contrary to what we observed as a numerical difference. Our and the aforementioned study differ in many aspects, which could at least partly explain the discrepancies. While we conducted a longitudinal study, the study by McGuire *et al*.^23^ had a cross-sectional design. While we assessed the impact of ppBMI on the concentrations of HMOs at specific times of lactation and adjusted for milk groups, McGuire *et al*.^23^ used maternal weight and BMI during lactation and HMO concentrations in HM collected anytime between 2 weeks and 5 months postpartum without such adjustments. Although purely speculative, a possible reason for the observed difference in OW mothers might be found in prolactin. OW/obese women have a lower prolactin response to suckling that may compromise their ability to produce milk^29^ and affect HM composition. Besides its role in initiation and maintenance of HM production, prolactin also mediates progressive changes in HM composition and recombinant human prolactin was shown to increase both neutral and acidic oligosaccharides^30,31^.

Interestingly and for the first time, we observed a relationship between several HMOs and the mode of delivery. We observe that among women delivering through C-section, the concentrations of 2′FL, 3′SL, and 6′GL were lower compared to those having a vaginal delivery. The magnitude of most differences was relatively small and mostly only seen at the first postpartum HM collection (day 2). We can only speculate on possible reasons for our observation. Parturition is triggered by several paracrine and autocrine events and fetal hormonal changes^32^. C-section was previously related with significantly lower levels of stress-associated hormone release when compared with vaginal delivery^33^. Whether or not such hormonal changes during parturition may influence maternal glycosylation and hence early HMO composition needs further investigation.

We observed several HMOs related with maternal parity, however with differences between PP and MP mothers of relatively small magnitude and only seen at single timepoints during early lactation (day 2 and 17). In our dataset, although not withstanding FDR correction for multiple comparisons, PP women showed lower concentrations of several HMOs, namely 3FL, 6′GL, LNFP-II and LNFP-V, while they had higher concentrations of other HMOs such as DSLNT and LNnT, compared to MP women. A recent publication from Azad and colleagues^24^ using data from the CHILD cohort is the only other study that has investigated the associations between parity and HMOs. They observed lower levels of LNnT, LNT and higher levels of 3FL in PP compared to MP women, contrary to what we observed. Theirs was a predominantly Caucasian population (73%) similar to ours, although HM samples were collected at 3–4 months postpartum and not earlier in lactation, when we observed most differences. While very little is reported on possible effects of parity for HMO composition, maternal parity seems to influence concentrations and daily productions of HM fat, nitrogen, lactose and total energy with PP women producing milk with a 23% higher fat concentration and their infants had a 30% higher intake of HM, compared to those with higher parity^34^.

In our observational longitudinal study the phenotypically determined fucosyltransferase FUT2 and FUT3 status together with time of lactation had the biggest impact on HMO variation. In comparison, other major maternal factors that we investigated had only marginal and time-restricted effects and many of the observed associations to maternal factors were lost upon correction for multiple comparisons. Still, our observations may indicate subtle changes in early post-partum physiology leading to changes in glycosylation pathways for some HMOs due to ppBMI, mode of delivery and possibly also parity. To date, our study is to our knowledge the largest European standardized and longitudinal milk collection study assessing HMO concentrations and basic maternal characteristics in a well controlled manner taking into account especially the time of lactation and milk groups. Limitations of our study include the use of self-reported maternal weight and height for calculation of ppBMI and the lack of maternal body compositional data as well as the missing HMO trajectories beyond the first 4 months of breastfeeding period. Future studies need to elucidate these observations and assess the physiological significance for the breastfed infant.

## Materials and Methods

### Study design and subjects enrollment

The study was conducted in seven countries across Europe (France, Italy, Norway, Portugal, Romania, Spain, and Sweden) as a longitudinal, observational, cohort in which HM as well as multiple maternal and infant parameters were collected at six time points post-partum (2 (0–3) d, 17 ± 3 d, 30 ± 3 d, 60 ± 5 d, 90 ± 5 d, and 120 ± 5 d). Institutional and local ethical boards of each centre approved the study. (France: ‘comité de protection des personnes sud-ouest et outre mer III’; Italy: Comitato Etico Ospedale di Circolo Fondazione Macchi; Norway: Regionale Komiteer for Medisinsk og Helsefaglig Forskningetikk; Portugal: Comissao de Etica do Centro Hospitalar Sao Joao, Comissao de Etica para a Saude do Hospital de Braga, Conselho de Administracao do Centro Hospitalar de Alto Ave; Romania: Comisia Locala de Etica, Spitalul Clinic Judetean de Urgenta Ilfov, Ethic Committee of Institutul Pentru Ocrotirea Namei si Copilului; Spain: Comité Ético de Investigación Clínica Del Hospital Clínico Universitario de Valencia; Sweden: Regionala etikprövningsnämnden Umeå). All methods were performed in accordance with the relevant local and institutional guidelines and regulations. The participants provided a written informed consent form to participate in the study after receiving explanations and having read and understood the purpose and the objectives of the study in their respective local languages. The study was registered at www.clinicaltrials.gov with the identifier NCT01894893. Pregnant women were recruited before delivery, generally during the last trimester of pregnancy. Inclusion criteria for this study were: (i) pregnant women between ages of 18 and 40 years; (ii) ppBMI between 19 and 29, inclusive; (iii) intention to breastfeed at least until 4 mo post-partum and (iv) agreed to the study protocol and signed informed consent form. Exclusion criteria for this study were: (i) currently participating in another trial; (ii) presenting conditions that contraindicate breastfeeding; (iii) medical conditions or on medications for conditions such as metabolic and cardiovascular abnormalities; (iv) dietary problems such as anorexia or bulimia and (v) subjects not able to comply to the study procedures. Dedicated, trained and certified research nurses and assistants collected all data for this study. Maternal data included: demography, anthropometry, medical history, history of dietary supplements, three-day food diaries. Infant data included: demography, anthropometry, history of medication use, body composition (one centre in France and one in Sweden), infant HM intake diary (three centres in France only).

### Standardized human milk sampling

HM sampling was standardized for all subjects. Milk was collected at 11h00 ± 2h00 using an electric breast pump (Medela Symphony, Switzerland). For each mother, HM was collected from the same breast for the entire study and mothers were requested to empty the breast manually in the previous feed. This collected single full breast milk samples were mixed and an aliquot of 5–10 mL for the first time point and 10–40 mL HM for all other time points was collected. The remainder of the HM was kept by the mother for future use. Each collected HM sample was transferred to freezing tubes, labelled with subject number and collection information, stored at −18 °C in the home freezer, transferred to the hospital for storage at −80 °C and then shipped on dry ice to the Nestlé Research Centre (Lausanne, Switzerland) for storage at −80 °C until analysis. The frozen HM samples were thawed once for aliquoting into 15 individual small volume fractions (0.2 mL to 2 mL) in separate polypropylene tubes dedicated to the different analyses.

### Assessment of maternal factors

Self-reported maternal pre-pregnancy weight and height was used to calculate the ppBMI. The ppBMI was calculated as weight (in kilograms) divided by height (in meters) squared (kg/m^2^). ppBMI was categorized based on the WHO guidelines into normal (NW: 18.5–24.9 kg/m^2^) and overweight (OW: 25.0–29.9 kg/m^2^). For parity we categorized mothers as either primiparous (PP) or multiparous (MP). For mode of delivery we categorized mothers as those giving birth via Cesarean section (C-section) versus vaginal delivery. Information on emergency or elective C-section was not collected.

### Quantification of HMOs

An HM aliquot was shipped on dry ice to Neotron Spa. (Italy) for HMO quantification by liquid chromatography with fluorescence detection after labelling with 2-aminobenzamide using the method of Austin & Benet^35^. Ten HMOs were quantified using genuine HMOs standards acquired from Elicityl (France) together with assessment of purity using quantitative nuclear magnetic resonance spectroscopy; 2′-fucosyllactose (2′FL), 3-fucosyllactose (3FL), Lacto-N-tetraose (LNT), Lacto-N-neotetraose (LNnT), 3′-sialyllactose (3′SL), 6′-sialyllactose (6′SL), Lacto-N-fucopentaose-I (LNFP-I), LNFP-V and Lacto-N-neofucopentaose (LNnFP). The remaining HMOs were quantified against maltotriose of known purity (Sigma-Aldrich, Germany) assuming equimolar response factors. The full name and abbreviations of the quantified HMOs and their structures are shown in Fig. 2.

### Assignment of milk groups

Based on the presence and absence of functional fucosyltransferases FUT2 and FUT3 human milk samples can be assigned to 4 distinct milk groups^21,36^. FUT2 attaches fucose through an α-1,2 linkage to the oligosaccharide and FUT3 attaches fucose through an α-1,3 or an α-1,4 linkage to oligosaccharides or lactose. Supplementary Fig. 1 illustrates the distribution of the principle determinant HMOs for milk groups 1, 2, 3 and 4. We assigned the HM to one of the four milk groups based on the concentrations of 2′FL (combined with lacto-N-fucopentaose-I (LNFP-I) as proxy for FUT2 activity and lacto-N-fucopentaose-II (LNFP-II) as proxy for FUT3 activity at visit 2. Milk samples with concentrations of 2′FL and LNFP-II above 25 mg/L were assigned to group 1, milk samples with concentrations of 2′FL below 25 mg/L and LNFP-II above 25 mg/L were assigned to milk group 2, milk samples with concentrations of 2′FL above 25 mg/L and LNFP-II below 25 mg/L were assigned to milk group 3, milk samples with concentrations of 2′FL and LNFP-II below 25 mg/L were assigned to milk group 4. The threshold of 25 mg/L was chosen based on the performance of the analytical method^35^.

### Statistical methods

All statistical analyses were done with the statistical software R v.3.2.3. Given the longitudinal nature of our data with repeat measures, a linear mixed model is used when assessing the variation of the different HMOs across time. To model the dependent variable HMO, all equations include visit and the fixed factor of milk group and as independent variables or covariates the different maternal factors. The within subject variability is taken in to consideration by setting subject as a random effect. The statistical models included available parameters that were expected or that were independently tested to affect the outcome measure. Throughout the manuscript we explore the following models:

$$\begin{array}{c}{\rm{ppBMI}}\\ HMO \sim ppBMI+visit+ppBMI:visit+milk\,group\\ {\rm{Parity}}\\ HMO \sim parity+visit+parity:visit+milk\,group+ppBMI\\ {\rm{Mode}}\,{\rm{of}}\,{\rm{delivery}}\\ {\rm{HMO}} \sim {\rm{delivery}}+{\rm{visit}}+{\rm{delivery}}:{\rm{visit}}+{\rm{milk}}\,{\rm{group}}+{\rm{parity}}+{\rm{ppBMI}}\end{array}$$

where *ppBMI* refers to the pre-pregnancy BMI (continuous variable), Where *visit* refers to the day of sample collection*, milk group* refers to milk groups based on HMO secretion, *parity* refers refers to the infant being multiparous or primiparous (categorical) and *delivery* refers to the mode of delivery of the infant. Adjustment for multiplicity was done according to Benjamini-Hochberg using the function p.adjust. Unadjusted and adjusted FDR (false discovery rate) p- and q- values are presented (adjustement per group comparison/scientific question) in order to have an indication of which differences are most important and robust. For mode of delivery (C-section versus Vaginal delivery) and parity (PM versus MP), we examined the distribution of milk sampling by day for visit 1, in order to exclude any effects skewing our observed associations with HMOs (Supplementary Figs 3–5).

For total HMO and individual HMOs a linear mixed model was created to look at the significance of milk groups and time. The model explains the total HMO (sum of 20 HMOs per subject and per timepoint) by the covariates milk group, age post-partum and the interaction between the two. Contrast estimates were used to calculate estimated differences between consecutive timepoints, as well as all timepoints compared to the first visit. An FDR adjustment was applied to the p-values to adjust for multiplicity (9 comparisons). Contrast estimates were used to calculate estimated differences between the milk groups. An FDR adjustment was applied to the p value to adjust for multiplicity (6 comparisons).

A similar linear mixed model was created to explain individual HMOs. An FDR adjustment was applied to the p value to adjust for multiplicity for all HMOs and all timepoint comparisons (20 × 9 comparisons) as well as for all HMOs and all milkgroups (20 × 6 comparisons).

## Supplementary information

Supplementary data
