# Supplementary material for: Impact of maternal characteristics on human milk oligosaccharide composition over the first 4 months of lactation in a cohort of healthy European mothers
Source: Sci Rep. 2019 Aug 13;9:11767. doi: 10.1038/s41598-019-48337-4 (PMC6692355; doi:10.1038/s41598-019-48337-4)
Supplement: Supplementary file 1 — Supplementary data [file 41598_2019_48337_MOESM1_ESM.docx]

**Supplementary data:**

**Impact of maternal characteristics on human milk oligosaccharide composition over the first 4 months of lactation in a cohort of healthy European mothers**

Tinu Mary Samuel^1+^, Aristea Binia^1+^, Carlos Antonio de Castro^2^, Sagar K. Thakkar^1^, Claude Billeaud^3^, Massimo Agosti^4^, Isam Al-Jashi^5^, Maria Jose Costeira^6^, Giovanna Marchini^7^, Cecilia Martínez-Costa^8^, Jean-Charles Picaud^9^, Tom Stiris^10^, Sylvia-Maria Stoicescu^11^, Mireille Vanpeé^7^, Magnus Domellöf^12^, Sean Austin^1^, Norbert Sprenger^1*^

^1^ Nestlé Research, Nestec Ltd., 1000 Lausanne 26, Switzerland

^2^ Nestlé Research, Nestec Ltd., 138567 Singapore

^3^ Hôpital des enfants, CHU Pellegrin, Bordeaux, France

^4^ Ospedale del Ponte, Varese, Italy

^5^ Al Jashi Isam Private Med. Practice, Bucharest, Romania

^6^ Instituto de Investigação em Ciências da Vida e Saúde, Braga, Portugal

^7^ Karolinska University Hospital, Stockholm

^8^ Hospital Clínico Universitario, University of Valencia, Valencia, Spain

^9^ Hôpital de la Croix Rousse, Lyon, France

^10^ Oslo University Hospital, Oslo, Norway

^11^ Polizu Hospital, Bucharest, Romania

^12^ Umeå University, Umeå, Sweden

^*^ Correspondance to: Norbert.Sprenger@rdls.nestle.com

^+^ these authors contributed equally to this work

**Supplementary Figure 1.**

**Supplementary Figure 1.** Definition of milk groups. Group 1 contains significant amounts of HMOs containing fucose residues linked through α-1,2, α-1,3 and α-1,4 linkages. Group 2 is typified by having significant amounts of HMOs with α-1,3 and α-1,4 linked fucose residues but low concentrations (or absence) of HMOs with α-1,2-linked fucose residues. Group 3 is typified by containing significant amounts of HMOs with α-1,2-linked fucose residues, and very low concentrations (or absence) of oligosaccharides with α-1,4-linked fucose residues. Group 4 is typified by having very low concentrations (or absence) of HMOs with α-1,2-linked fucose residues and α-1,4-linked fucose residues.

**Supplementary Figure 2.**


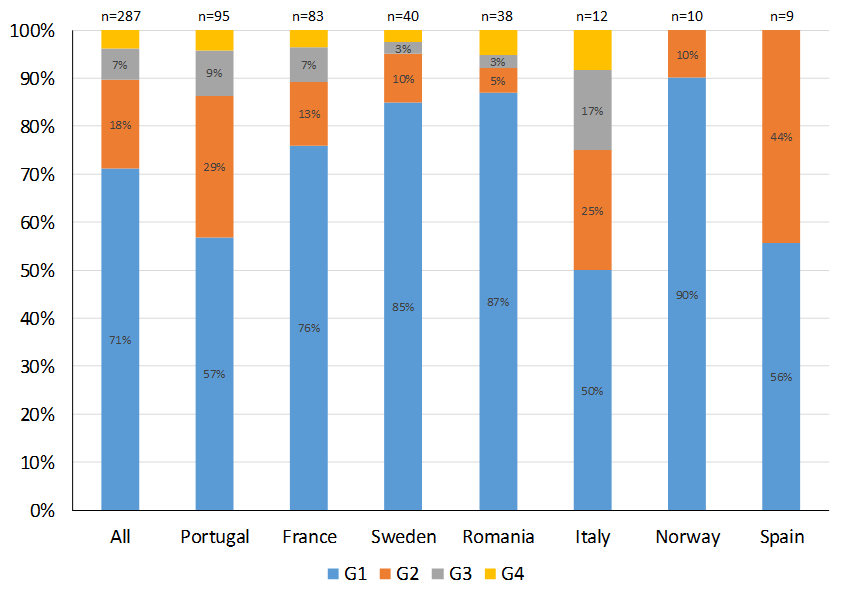


**Supplementary Figure 2.** Distribution of milk groups by country.

**Supplementary Figure 3.**

**Supplementary Figure 3.** Distribution of HM samples collected at visit 1 by day post-partum (day 1 to day 9) and by mode of delivery (A) and parity (B).

**Supplementary Figure 4.**
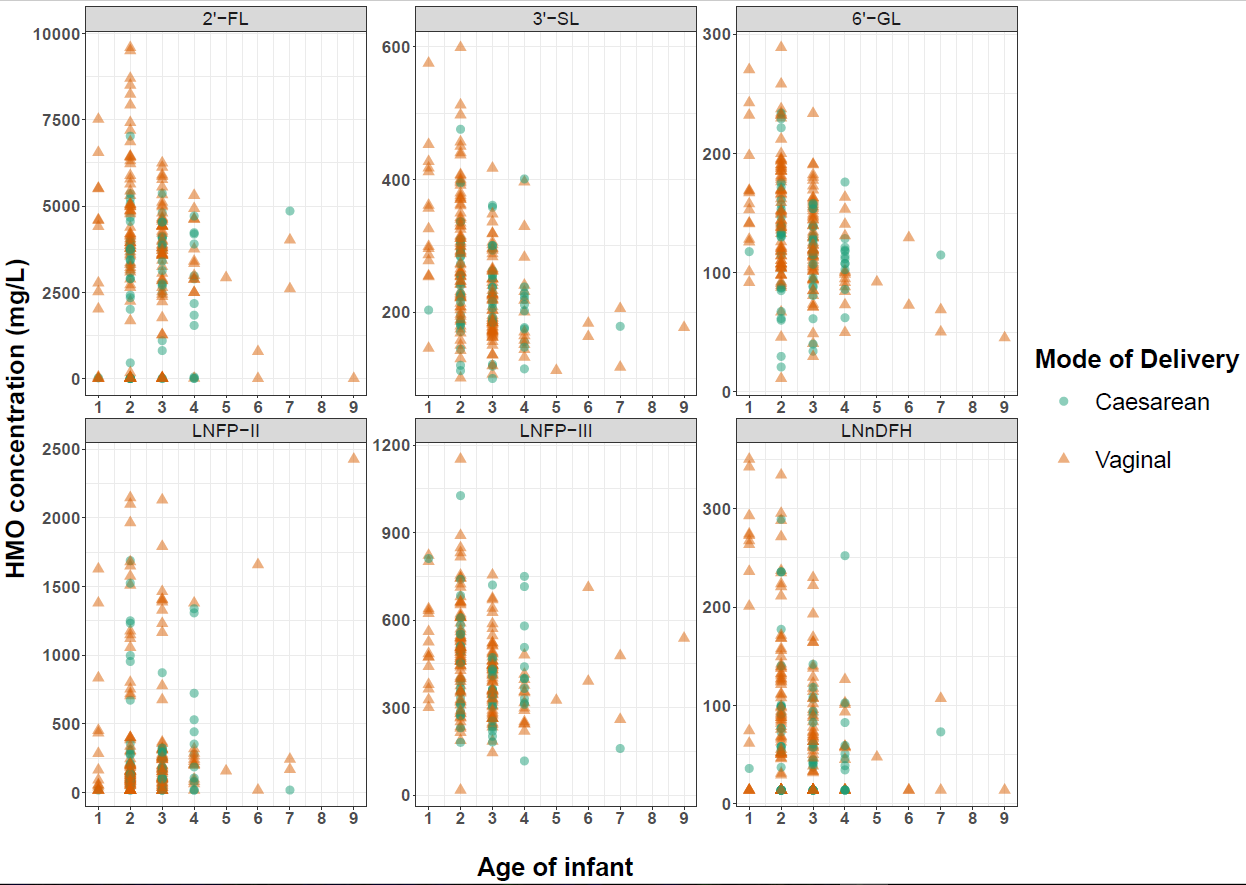


**Supplementary Figure 4.** Distribution of HMO concentrations for visit 1 (day 1 to day 9) by on mode of delivery.

**Supplementary Figure 5.**


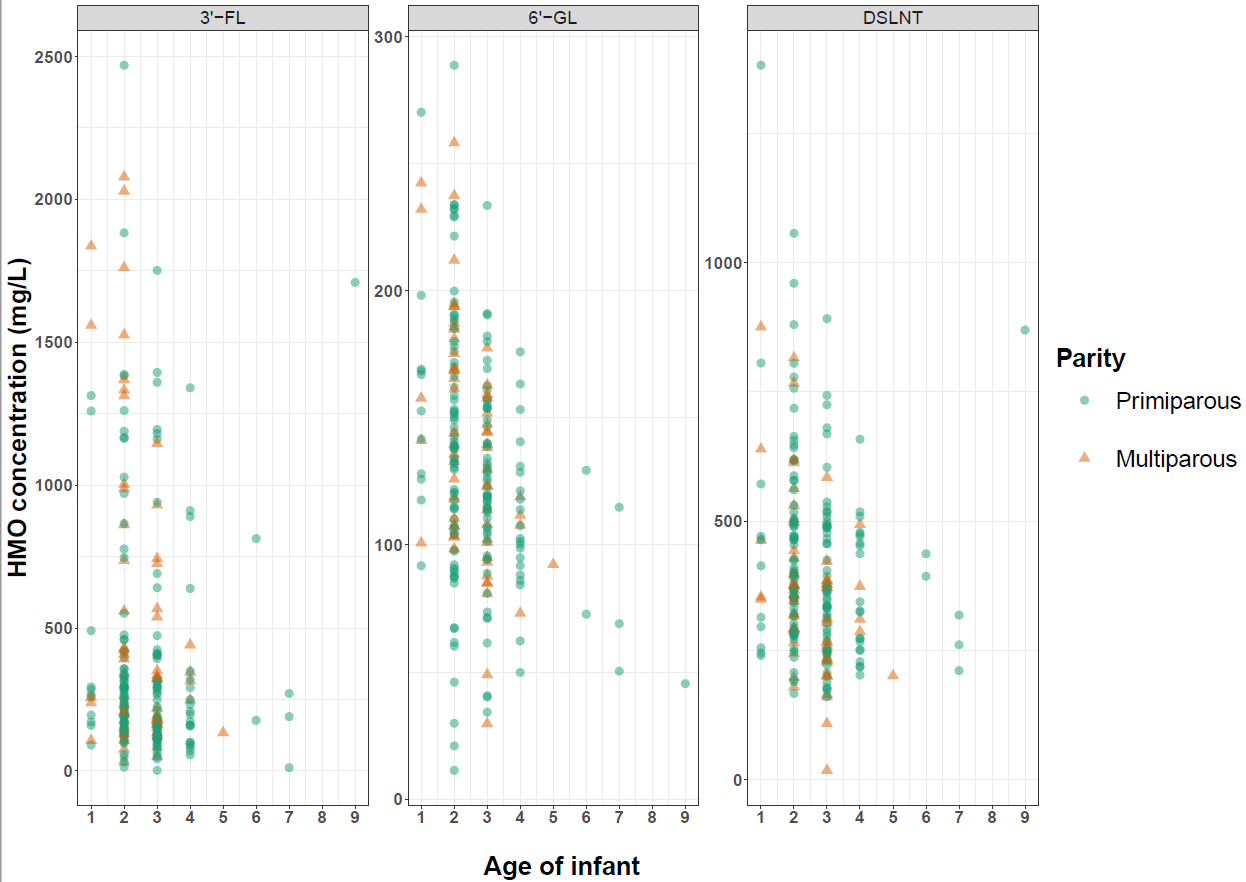


**Supplementary Figure 5.** Distribution of HMO concentrations for visit 1 (day 1 to day 9) by maternal parity.

**Supplementary Table 1.** Detailed information on reasons for dropping out of the study.

| **Reason for drop out** | **category** | **n** |
| --- | --- | --- |
| The mother withdrew without explanation | The mother withdrew without explanation | 37 |
| Agalactorrhea | Agalactorrhea | 50 |
| Consent withdrawn | Consent withdrawn | 10 |
| Stop breastfeeding | Stop breastfeeding | 1 |
| Stop breastfeeding to return to work | Stop breastfeeding | 1 |
| Not respecting inclusion criteria | Outside inclusion criteria | 1 |
| ppBMI outside inclusion criteria | Outside inclusion criteria | 1 |
| Don't think to breastfeed for 4 months | Other | 1 |
| Fear of Pea-Pod | Other | 1 |
| Living far from hospital, too much effort | Other | 1 |
| Mother had a full time job and gave other diet to infant | Other | 1 |
| Parents did not want to participate anymore | Other | 1 |
| The mother continues breastfeeding the older child | Other | 1 |
| The mother could not come for V4, then for V5 and V6 and infant started on formula milk | Other | 1 |
| The mother did have problem to start with breastfeeding the baby | Other | 1 |
| The mother had not enough energy | Other | 1 |
| Unable to come to last visit | Other | 1 |
| Maternal infections and infestations | Maternal infections and infestations | 6 |
| Difficulties to breastfeed. Maternal breast infection. Infant on formula milk. | Maternal disorder | 1 |
| Gestational diabetes and hypothyroidism | Maternal disorder | 1 |
| Hypothyroidism recorded | Maternal disorder | 1 |
| Maternal gastrointestinal disorders | Maternal disorder | 1 |
| Maternal pregnancy, puerperium and perinatal conditions | Maternal disorder | 1 |
| Maternal renal and urinary disorders | Maternal disorder | 1 |
| Feeding the infant formula for more than 7 days in a row | Infant on formula milk | 4 |
| Infant on formula milk | Infant on formula milk | 3 |
| Infant >7 days on formula milk | Infant on formula milk | 2 |
| Infant on formula milk from one week, twice daily | Infant on formula milk | 1 |
| Infant on formula milk on a daily basis | Infant on formula milk | 1 |
| Infant received formula milk for >7 days in a row due to breastfeeding difficulty | Infant on formula milk | 1 |
| Poor infant weight gain and introduction of formula milk | Infant on formula milk | 1 |
| Infant respiratory, thoracic and mediastinal disorders | Infant illness | 2 |
| Congenital, familial and genetic disorders | Infant disorder | 1 |
| Delivery in another hospital | Infant disorder | 1 |
| Hypogalactorrhea and cow milk protein allergy | Infant disorder | 1 |
| Infant infections and infestations | Infant disorder | 1 |
| Infant metabolism and nutrition disorders | Infant disorder | 1 |
| Infant musculoskeletal and connective tissue disorders | Infant disorder | 1 |

**Supplementary Table 2.** HMO concentrations over first 4 months of lactation in mg/L.

| **Abbr** | **HMO** | **visit** | **days postpartum** | **N** | **mean** | **SD** | **median** | **q1 (25%)** | **q3 (75%)** | **min** | **max** |
| --- | --- | --- | --- | --- | --- | --- | --- | --- | --- | --- | --- |
| 2'FL | 2'-Fucosyllactose | V1 | 2 | 207 | 3691 | 1941 | 3798 | 2747 | 4745 | 13 | 9589 |
|  | 2'-Fucosyllactose | V2 | 17 | 230 | 2627 | 1028 | 2630 | 1991 | 3284 | 16 | 5849 |
|  | 2'-Fucosyllactose | V3 | 30 | 205 | 2450 | 935 | 2428 | 1901 | 3081 | 17 | 4485 |
|  | 2'-Fucosyllactose | V4 | 60 | 188 | 2075 | 840 | 2005 | 1546 | 2603 | 19 | 4027 |
|  | 2'-Fucosyllactose | V5 | 90 | 179 | 1819 | 739 | 1728 | 1330 | 2351 | 19 | 3820 |
|  | 2'-Fucosyllactose | V6 | 120 | 169 | 1625 | 670 | 1559 | 1184 | 2073 | 16 | 3608 |
| 3FL | 3-Fucosyllactose | V1 | 2 | 236 | 422 | 453 | 249 | 155 | 424 | 11 | 2469 |
|  | 3-Fucosyllactose | V2 | 17 | 289 | 594 | 554 | 368 | 236 | 698 | 40 | 2638 |
|  | 3-Fucosyllactose | V3 | 30 | 260 | 720 | 608 | 485 | 300 | 872 | 57 | 2922 |
|  | 3-Fucosyllactose | V4 | 60 | 242 | 970 | 692 | 769 | 482 | 1255 | 78 | 3279 |
|  | 3-Fucosyllactose | V5 | 90 | 233 | 1140 | 777 | 937 | 636 | 1526 | 85 | 5716 |
|  | 3-Fucosyllactose | V6 | 120 | 223 | 1209 | 717 | 1074 | 662 | 1644 | 109 | 3653 |
| 6'GL | 6'-Galactosyllactose | V1 | 2 | 237 | 132 | 47 | 129 | 102 | 159 | 11 | 289 |
|  | 6'-Galactosyllactose | V2 | 17 | 288 | 40 | 31 | 35 | 28 | 44 | 14 | 434 |
|  | 6'-Galactosyllactose | V3 | 30 | 261 | 26 | 10 | 24 | 19 | 29 | 7 | 89 |
|  | 6'-Galactosyllactose | V4 | 60 | 240 | 18 | 9 | 16 | 13 | 20 | 7 | 74 |
|  | 6'-Galactosyllactose | V5 | 90 | 226 | 14 | 7 | 12 | 10 | 17 | 7 | 60 |
|  | 6'-Galactosyllactose | V6 | 120 | 208 | 13 | 11 | 11 | 9 | 14 | 6 | 147 |
| 3'SL | 3'-Sialyllactose | V1 | 2 | 237 | 254 | 90 | 240 | 185 | 302 | 100 | 599 |
|  | 3'-Sialyllactose | V2 | 17 | 290 | 149 | 38 | 144 | 121 | 168 | 74 | 322 |
|  | 3'-Sialyllactose | V3 | 30 | 261 | 141 | 35 | 135 | 116 | 161 | 79 | 260 |
|  | 3'-Sialyllactose | V4 | 60 | 243 | 129 | 31 | 126 | 108 | 147 | 72 | 248 |
|  | 3'-Sialyllactose | V5 | 90 | 234 | 130 | 35 | 124 | 106 | 147 | 64 | 272 |
|  | 3'-Sialyllactose | V6 | 120 | 224 | 132 | 38 | 126 | 104 | 155 | 63 | 314 |
| 6'SL | 6'-Sialyllactose | V1 | 2 | 237 | 543 | 168 | 535 | 425 | 630 | 168 | 1096 |
|  | 6'-Sialyllactose | V2 | 17 | 289 | 649 | 189 | 636 | 515 | 776 | 166 | 1310 |
|  | 6'-Sialyllactose | V3 | 30 | 261 | 465 | 162 | 452 | 348 | 556 | 65 | 1101 |
|  | 6'-Sialyllactose | V4 | 60 | 243 | 231 | 101 | 215 | 161 | 283 | 53 | 844 |
|  | 6'-Sialyllactose | V5 | 90 | 234 | 151 | 87 | 133 | 99 | 177 | 32 | 953 |
|  | 6'-Sialyllactose | V6 | 120 | 223 | 101 | 72 | 88 | 64 | 122 | 22 | 899 |
| LDFT | Lactodifucosyllactose | V1 | 2 | 186 | 607 | 558 | 424 | 230 | 741 | 51 | 3297 |
|  | Lactodifucosyllactose | V2 | 17 | 221 | 349 | 379 | 229 | 160 | 374 | 46 | 2996 |
|  | Lactodifucosyllactose | V3 | 30 | 198 | 277 | 231 | 227 | 153 | 305 | 50 | 1683 |
|  | Lactodifucosyllactose | V4 | 60 | 182 | 280 | 155 | 240 | 188 | 327 | 48 | 1109 |
|  | Lactodifucosyllactose | V5 | 90 | 175 | 273 | 131 | 254 | 188 | 335 | 46 | 824 |
|  | Lactodifucosyllactose | V6 | 120 | 168 | 269 | 126 | 246 | 194 | 323 | 51 | 790 |
| LNT | Lacto-N-Tetraose | V1 | 2 | 237 | 912 | 802 | 685 | 389 | 1213 | 22 | 6714 |
|  | Lacto-N-Tetraose | V2 | 17 | 290 | 1213 | 720 | 1080 | 708 | 1485 | 125 | 5361 |
|  | Lacto-N-Tetraose | V3 | 30 | 261 | 1009 | 591 | 876 | 632 | 1288 | 119 | 4012 |
|  | Lacto-N-Tetraose | V4 | 60 | 242 | 700 | 416 | 629 | 428 | 827 | 121 | 2894 |
|  | Lacto-N-Tetraose | V5 | 90 | 234 | 599 | 400 | 493 | 350 | 747 | 86 | 2933 |
|  | Lacto-N-Tetraose | V6 | 120 | 222 | 526 | 358 | 458 | 293 | 641 | 90 | 3026 |
| LNnT | Lacto-N-Neotetraose | V1 | 2 | 237 | 307 | 132 | 294 | 214 | 380 | 48 | 699 |
|  | Lacto-N-Neotetraose | V2 | 17 | 285 | 177 | 97 | 165 | 102 | 234 | 26 | 597 |
|  | Lacto-N-Neotetraose | V3 | 30 | 255 | 153 | 80 | 142 | 87 | 203 | 27 | 425 |
|  | Lacto-N-Neotetraose | V4 | 60 | 232 | 128 | 80 | 111 | 69 | 172 | 25 | 464 |
|  | Lacto-N-Neotetraose | V5 | 90 | 218 | 108 | 67 | 92 | 59 | 139 | 25 | 399 |
|  | Lacto-N-Neotetraose | V6 | 120 | 208 | 98 | 62 | 83 | 49 | 121 | 24 | 298 |
| LNFP-I | Lacto-N-Fucosylpentaose-I | V1 | 2 | 187 | 1928 | 903 | 1848 | 1234 | 2520 | 27 | 4311 |
|  | Lacto-N-Fucosylpentaose-I | V2 | 17 | 224 | 1431 | 798 | 1270 | 800 | 2001 | 50 | 3756 |
|  | Lacto-N-Fucosylpentaose-I | V3 | 30 | 201 | 1071 | 627 | 965 | 575 | 1497 | 29 | 3307 |
|  | Lacto-N-Fucosylpentaose-I | V4 | 60 | 183 | 611 | 423 | 516 | 282 | 802 | 31 | 2069 |
|  | Lacto-N-Fucosylpentaose-I | V5 | 90 | 175 | 469 | 373 | 376 | 192 | 612 | 28 | 2063 |
|  | Lacto-N-Fucosylpentaose-I | V6 | 120 | 165 | 384 | 316 | 312 | 160 | 500 | 28 | 1913 |
| LNFP-II | Lacto-N-Fucosylpentaose-II | V1 | 2 | 202 | 422 | 518 | 197 | 107 | 380 | 37 | 2427 |
|  | Lacto-N-Fucosylpentaose-II | V2 | 17 | 260 | 595 | 630 | 351 | 198 | 668 | 35 | 3602 |
|  | Lacto-N-Fucosylpentaose-II | V3 | 30 | 230 | 549 | 532 | 343 | 212 | 635 | 53 | 2623 |
|  | Lacto-N-Fucosylpentaose-II | V4 | 60 | 213 | 474 | 402 | 331 | 217 | 587 | 36 | 2274 |
|  | Lacto-N-Fucosylpentaose-II | V5 | 90 | 209 | 433 | 332 | 335 | 207 | 534 | 42 | 1715 |
|  | Lacto-N-Fucosylpentaose-II | V6 | 120 | 199 | 394 | 284 | 301 | 215 | 475 | 44 | 1554 |
| LNFP-III | Lacto-N-Fucosylpentaose-III | V1 | 2 | 236 | 445 | 166 | 432 | 322 | 527 | 117 | 1152 |
|  | Lacto-N-Fucosylpentaose-III | V2 | 17 | 289 | 320 | 141 | 307 | 236 | 377 | 80 | 1751 |
|  | Lacto-N-Fucosylpentaose-III | V3 | 30 | 260 | 311 | 98 | 303 | 255 | 362 | 97 | 952 |
|  | Lacto-N-Fucosylpentaose-III | V4 | 60 | 242 | 358 | 110 | 349 | 292 | 414 | 81 | 1196 |
|  | Lacto-N-Fucosylpentaose-III | V5 | 90 | 233 | 353 | 92 | 349 | 280 | 416 | 126 | 680 |
|  | Lacto-N-Fucosylpentaose-III | V6 | 120 | 223 | 339 | 92 | 333 | 277 | 404 | 134 | 568 |
| LNFP-V | Lacto-N-Fucosylpentaose-V | V1 | 2 | 158 | 108 | 103 | 51 | 35 | 174 | 24 | 393 |
|  | Lacto-N-Fucosylpentaose-V | V2 | 17 | 243 | 124 | 117 | 67 | 44 | 190 | 24 | 515 |
|  | Lacto-N-Fucosylpentaose-V | V3 | 30 | 220 | 112 | 99 | 66 | 46 | 145 | 24 | 427 |
|  | Lacto-N-Fucosylpentaose-V | V4 | 60 | 206 | 91 | 72 | 61 | 41 | 118 | 24 | 412 |
|  | Lacto-N-Fucosylpentaose-V | V5 | 90 | 195 | 85 | 66 | 59 | 41 | 105 | 26 | 360 |
|  | Lacto-N-Fucosylpentaose-V | V6 | 120 | 186 | 76 | 57 | 54 | 39 | 96 | 24 | 325 |
| LNnFP-V | Lacto-N-Neofucosylpentaose-V | V1 | 2 | 72 | 37 | 16 | 32 | 25 | 40 | 20 | 92 |
|  | Lacto-N-Neofucosylpentaose-V | V2 | 17 | 58 | 28 | 10 | 26 | 21 | 29 | 19 | 60 |
|  | Lacto-N-Neofucosylpentaose-V | V3 | 30 | 51 | 28 | 9 | 25 | 22 | 31 | 19 | 54 |
|  | Lacto-N-Neofucosylpentaose-V | V4 | 60 | 51 | 31 | 12 | 28 | 24 | 33 | 19 | 83 |
|  | Lacto-N-Neofucosylpentaose-V | V5 | 90 | 42 | 28 | 8 | 26 | 22 | 29 | 19 | 57 |
|  | Lacto-N-Neofucosylpentaose-V | V6 | 120 | 32 | 28 | 10 | 24 | 21 | 30 | 19 | 65 |
| LSTb | Sialyllacto-N-Tetraose b | V1 | 2 | 234 | 79 | 40 | 70 | 54 | 94 | 15 | 276 |
|  | Sialyllacto-N-Tetraose b | V2 | 17 | 289 | 80 | 40 | 72 | 53 | 97 | 16 | 323 |
|  | Sialyllacto-N-Tetraose b | V3 | 30 | 260 | 77 | 38 | 70 | 50 | 96 | 16 | 230 |
|  | Sialyllacto-N-Tetraose b | V4 | 60 | 237 | 64 | 33 | 58 | 42 | 77 | 17 | 203 |
|  | Sialyllacto-N-Tetraose b | V5 | 90 | 228 | 57 | 31 | 50 | 34 | 69 | 16 | 176 |
|  | Sialyllacto-N-Tetraose b | V6 | 120 | 218 | 50 | 28 | 43 | 31 | 62 | 14 | 161 |
| LSTc | Sialyllacto-N-Tetraose c | V1 | 2 | 237 | 497 | 218 | 459 | 338 | 602 | 123 | 1429 |
|  | Sialyllacto-N-Tetraose c | V2 | 17 | 290 | 258 | 128 | 231 | 167 | 315 | 35 | 887 |
|  | Sialyllacto-N-Tetraose c | V3 | 30 | 261 | 148 | 72 | 136 | 99 | 179 | 9 | 479 |
|  | Sialyllacto-N-Tetraose c | V4 | 60 | 243 | 70 | 47 | 61 | 42 | 85 | 11 | 480 |
|  | Sialyllacto-N-Tetraose c | V5 | 90 | 234 | 44 | 42 | 35 | 23 | 52 | 0 | 503 |
|  | Sialyllacto-N-Tetraose c | V6 | 120 | 224 | 29 | 37 | 22 | 15 | 34 | 0 | 508 |
| LNDFH-I | Lacto-N-Difucosylhexaose-I | V1 | 2 | 172 | 1232 | 519 | 1172 | 852 | 1484 | 12 | 3347 |
|  | Lacto-N-Difucosylhexaose-I | V2 | 17 | 209 | 1275 | 548 | 1203 | 955 | 1559 | 12 | 3583 |
|  | Lacto-N-Difucosylhexaose-I | V3 | 30 | 184 | 1105 | 452 | 1029 | 835 | 1347 | 11 | 3501 |
|  | Lacto-N-Difucosylhexaose-I | V4 | 60 | 169 | 842 | 327 | 801 | 655 | 1029 | 12 | 1918 |
|  | Lacto-N-Difucosylhexaose-I | V5 | 90 | 161 | 719 | 285 | 695 | 537 | 865 | 103 | 1793 |
|  | Lacto-N-Difucosylhexaose-I | V6 | 120 | 156 | 619 | 241 | 598 | 465 | 753 | 61 | 1485 |
| LNnDFH | Lacto-N-Neodifucosylhexaose | V1 | 2 | 163 | 113 | 73 | 91 | 58 | 138 | 29 | 350 |
|  | Lacto-N-Neodifucosylhexaose | V2 | 17 | 88 | 63 | 69 | 44 | 36 | 69 | 29 | 612 |
|  | Lacto-N-Neodifucosylhexaose | V3 | 30 | 58 | 58 | 53 | 47 | 32 | 67 | 28 | 398 |
|  | Lacto-N-Neodifucosylhexaose | V4 | 60 | 75 | 55 | 31 | 45 | 36 | 67 | 28 | 224 |
|  | Lacto-N-Neodifucosylhexaose | V5 | 90 | 56 | 57 | 29 | 47 | 35 | 70 | 28 | 142 |
|  | Lacto-N-Neodifucosylhexaose | V6 | 120 | 45 | 62 | 31 | 51 | 37 | 83 | 29 | 152 |
| DSLNT | Disialyllacto-N-Tetraose | V1 | 2 | 236 | 405 | 178 | 369 | 279 | 489 | 108 | 1382 |
|  | Disialyllacto-N-Tetraose | V2 | 17 | 290 | 385 | 164 | 358 | 272 | 462 | 64 | 1010 |
|  | Disialyllacto-N-Tetraose | V3 | 30 | 261 | 290 | 135 | 266 | 189 | 355 | 53 | 992 |
|  | Disialyllacto-N-Tetraose | V4 | 60 | 241 | 169 | 83 | 151 | 107 | 207 | 50 | 611 |
|  | Disialyllacto-N-Tetraose | V5 | 90 | 231 | 136 | 72 | 119 | 86 | 168 | 40 | 608 |
|  | Disialyllacto-N-Tetraose | V6 | 120 | 222 | 121 | 60 | 105 | 79 | 150 | 36 | 378 |
| MFLNH-III | Fucosyllacto-N-Hexaose-III | V1 | 2 | 216 | 201 | 155 | 160 | 94 | 256 | 36 | 977 |
|  | Fucosyllacto-N-Hexaose-III | V2 | 17 | 289 | 416 | 208 | 373 | 287 | 487 | 58 | 1490 |
|  | Fucosyllacto-N-Hexaose-III | V3 | 30 | 260 | 358 | 192 | 319 | 239 | 410 | 66 | 1373 |
|  | Fucosyllacto-N-Hexaose-III | V4 | 60 | 243 | 208 | 127 | 177 | 126 | 243 | 56 | 827 |
|  | Fucosyllacto-N-Hexaose-III | V5 | 90 | 231 | 143 | 94 | 117 | 79 | 170 | 35 | 595 |
|  | Fucosyllacto-N-Hexaose-III | V6 | 120 | 210 | 113 | 94 | 85 | 64 | 124 | 35 | 820 |
| DFLNHa | Difucosyllacto-N-Hexaose-a | V1 | 2 | 173 | 162 | 96 | 143 | 97 | 204 | 33 | 577 |
|  | Difucosyllacto-N-Hexaose-a | V2 | 17 | 221 | 278 | 163 | 238 | 172 | 343 | 33 | 1087 |
|  | Difucosyllacto-N-Hexaose-a | V3 | 30 | 195 | 227 | 147 | 185 | 127 | 291 | 39 | 807 |
|  | Difucosyllacto-N-Hexaose-a | V4 | 60 | 160 | 120 | 97 | 98 | 54 | 145 | 34 | 607 |
|  | Difucosyllacto-N-Hexaose-a | V5 | 90 | 107 | 98 | 80 | 72 | 52 | 104 | 33 | 515 |
|  | Difucosyllacto-N-Hexaose-a | V6 | 120 | 82 | 75 | 52 | 60 | 43 | 81 | 33 | 270 |

**Supplementary Table 3.** Concentrations of signifificantly different HMO in overweight and normal weight women across lactation.

| Time of lactation | HMO | Overweight women (OW) (mg/l) * | Normal weight women (NW) (mg/l) * | Mean difference(mg/l) | *P*-value | *q-value* |
| --- | --- | --- | --- | --- | --- | --- |
| Day 2 | 3’SL | 254 (203, 328) | 232 (184, 299) | 22 | <0.001 | 0.002 |
|  | 6’GL | 141 (127, 179) | 120 (95, 154) | 29.5 | <0.001 | <0.001 |
|  | DSLNT | 407 (322, 570) | 361 (269, 473) | 46 | 0.048 | 0.42 |
|  | LNnT | 262 (182, 324) | 307 (219, 404) | 45 | 0.02 | 0.42 |
| Day 17 | 6’SL | 710 (562, 797) | 628 (506, 762) | 82 | 0.03 | 0.42 |
| Day 30 | LNT | 845 (515, 1250) | 882 (638, 1299) | 37 | 0.04 | 0.42 |
| Day 60 | LNFP-V | 48 (12, 94) | 52 (32, 94) | 10 | 0.008 | 0.32 |
| Day 90 | LNT | 480 (281, 720) | 494 (361, 759) | 14 | 0.04 | 0.42 |
|  | LNFP-V | 53 (16, 91) | 51 (32, 93) | 2 | 0.039 | 0.42 |
| Day 120 | LNFP-V | 48 (26, 77) | 46 (29, 78) | 2 | 0.04 | 0.42 |

*Values are median (25^th^, 75^th^ percentile)

**Supplementary Table 4**. Concentrations of signifificantly different HMO among women delivering via C-section and vaginal delivery across lactation.

| Time of lactation | HMO | C-section  (CS) (mg/l) * | vaginal delivery  (VB) (mg/l) * | Mean difference (mg/l) | *P*-value | *q-value* |
| --- | --- | --- | --- | --- | --- | --- |
| Day 2 | LNT | 713 (474, 1062) | 679 (360, 1240) | 33 | 0.02 | 0.41 |
|  | 2'FL | 3267 (1543, 4106) | 3719 (1746, 4811) | 517 | <0.0001 | 0.0002 |
|  | 3’SL | 228 (185, 289) | 247 (185, 314) | 26 | 0.0003 | 0.012 |
|  | 6’GL | 119 (90, 139) | 132 (104, 167) | 17 | <0.0001 | 0.0009 |
|  | LNFP III | 367 (307, 493) | 445 (329, 538) | 41 | 0.024 | 0.41 |
|  | LNnDFH | 51 (14, 91) | 67 (14, 125) | 22 | 0.024 | 0.41 |
|  | LNFP II | 155 (94, 323) | 161 (72, 316) | 50 | 0.028 | 0.41 |
| Day 30 | 6’SL | 494 (366, 563) | 441 (342, 554) | 39 | 0.019 | 0.41 |

*Values are median (25^th^, 75^th^ percentile)

**Supplementary Table 5.** Concentrations of signifificantly different HMO among primiparous and multiparous women across lactation.

| Time of lactation | HMO | Primiparous (PP)  (mg/l) * | Multiparous (MP)  (mg/l) * | Mean difference  (mg/l) | *P*-value | *q-value* |
| --- | --- | --- | --- | --- | --- | --- |
| Day 2 | DSLNT | 375 (284,493) | 351 (263, 405) | 56 | 0.003 | 0.16 |
|  | 6’GL | 126 (100, 154) | 137 (106, 169) | 10 | 0.001 | 0.16 |
|  | 3FL | 237 (147, 397) | 290 (169, 728) | 136 | 0.039 | 0.85 |
| Day 17 | LNnT | 172 (102, 246) | 131 (89, 197) | 34 | 0.011 | 0.44 |
|  | LNFP II | 287 (123, 507) | 424 (234, 1050) | 202 | 0.044 | 0.85 |
|  | LNFP V | 53 (32, 108) | 71 (45, 184) | 27 | 0.044 | 0.85 |

*Values are median (25^th^, 75^th^ percentile)

**Supplementary Table 6.** Statistical evaluation of HMO differences among the 4 milk groups.

|  | HMO | comparison | estimate | p-value | q-value |
| --- | --- | --- | --- | --- | --- |
| 1 | DFLNHa | Group 1 vs Group 2 | 106 | 0.0000 | 0.0000 |
| 2 | DFLNHa | Group 1 vs Group 3 | -170 | 0.0000 | 0.0000 |
| 3 | DFLNHa | Group 1 vs Group 4 | 107 | 0.0000 | 0.0000 |
| 4 | DFLNHa | Group 2 vs Group 3 | -277 | 0.0000 | 0.0000 |
| 5 | DFLNHa | Group 2 vs Group 4 | 1 | 0.9606 | 0.9852 |
| 6 | DFLNHa | Group 3 vs Group 4 | 278 | 0.0000 | 0.0000 |
| 7 | 2'FL | Group 1 vs Group 2 | 2345 | 0.0000 | 0.0000 |
| 8 | 2'FL | Group 1 vs Group 3 | -595 | 0.0014 | 0.0026 |
| 9 | 2'FL | Group 1 vs Group 4 | 2363 | 0.0000 | 0.0000 |
| 10 | 2'FL | Group 2 vs Group 3 | -2940 | 0.0000 | 0.0000 |
| 11 | 2'FL | Group 2 vs Group 4 | 18 | 0.9486 | 0.9813 |
| 12 | 2'FL | Group 3 vs Group 4 | 2958 | 0.0000 | 0.0000 |
| 13 | LNFP-V | Group 1 vs Group 2 | -160 | 0.0000 | 0.0000 |
| 14 | LNFP-V | Group 1 vs Group 3 | 11 | 0.2944 | 0.4014 |
| 15 | LNFP-V | Group 1 vs Group 4 | -154 | 0.0000 | 0.0000 |
| 16 | LNFP-V | Group 2 vs Group 3 | 172 | 0.0000 | 0.0000 |
| 17 | LNFP-V | Group 2 vs Group 4 | 7 | 0.6671 | 0.7772 |
| 18 | LNFP-V | Group 3 vs Group 4 | -165 | 0.0000 | 0.0000 |
| 19 | LNFP-II | Group 1 vs Group 2 | -909 | 0.0000 | 0.0000 |
| 20 | LNFP-II | Group 1 vs Group 3 | 273 | 0.0000 | 0.0000 |
| 21 | LNFP-II | Group 1 vs Group 4 | 240 | 0.0033 | 0.0058 |
| 22 | LNFP-II | Group 2 vs Group 3 | 1182 | 0.0000 | 0.0000 |
| 23 | LNFP-II | Group 2 vs Group 4 | 1149 | 0.0000 | 0.0000 |
| 24 | LNFP-II | Group 3 vs Group 4 | -33 | 0.7336 | 0.8305 |
| 25 | DSLNT | Group 1 vs Group 2 | -54 | 0.0004 | 0.0008 |
| 26 | DSLNT | Group 1 vs Group 3 | -9 | 0.6942 | 0.7934 |
| 27 | DSLNT | Group 1 vs Group 4 | -182 | 0.0000 | 0.0000 |
| 28 | DSLNT | Group 2 vs Group 3 | 45 | 0.0787 | 0.1226 |
| 29 | DSLNT | Group 2 vs Group 4 | -129 | 0.0001 | 0.0003 |
| 30 | DSLNT | Group 3 vs Group 4 | -174 | 0.0000 | 0.0000 |
| 31 | LNnFP | Group 1 vs Group 2 | -5 | 0.0000 | 0.0000 |
| 32 | LNnFP | Group 1 vs Group 3 | 2 | 0.1324 | 0.1962 |
| 33 | LNnFP | Group 1 vs Group 4 | 3 | 0.1761 | 0.2547 |
| 34 | LNnFP | Group 2 vs Group 3 | 7 | 0.0001 | 0.0001 |
| 35 | LNnFP | Group 2 vs Group 4 | 8 | 0.0009 | 0.0019 |
| 36 | LNnFP | Group 3 vs Group 4 | 1 | 0.8285 | 0.9038 |
| 37 | LSTb | Group 1 vs Group 2 | -28 | 0.0000 | 0.0000 |
| 38 | LSTb | Group 1 vs Group 3 | 2 | 0.7555 | 0.8473 |
| 39 | LSTb | Group 1 vs Group 4 | -37 | 0.0000 | 0.0001 |
| 40 | LSTb | Group 2 vs Group 3 | 30 | 0.0000 | 0.0001 |
| 41 | LSTb | Group 2 vs Group 4 | -9 | 0.3509 | 0.4731 |
| 42 | LSTb | Group 3 vs Group 4 | -39 | 0.0002 | 0.0005 |
| 43 | 6'SL | Group 1 vs Group 2 | 9 | 0.5814 | 0.6976 |
| 44 | 6'SL | Group 1 vs Group 3 | -78 | 0.0022 | 0.0041 |
| 45 | 6'SL | Group 1 vs Group 4 | -8 | 0.8105 | 0.8922 |
| 46 | 6'SL | Group 2 vs Group 3 | -88 | 0.0023 | 0.0042 |
| 47 | 6'SL | Group 2 vs Group 4 | -18 | 0.6347 | 0.7467 |
| 48 | 6'SL | Group 3 vs Group 4 | 70 | 0.0971 | 0.1494 |
| 49 | MFLNH-III | Group 1 vs Group 2 | -91 | 0.0000 | 0.0000 |
| 50 | MFLNH-III | Group 1 vs Group 3 | -132 | 0.0000 | 0.0000 |
| 51 | MFLNH-III | Group 1 vs Group 4 | -350 | 0.0000 | 0.0000 |
| 52 | MFLNH-III | Group 2 vs Group 3 | -42 | 0.1574 | 0.2304 |
| 53 | MFLNH-III | Group 2 vs Group 4 | -259 | 0.0000 | 0.0000 |
| 54 | MFLNH-III | Group 3 vs Group 4 | -218 | 0.0000 | 0.0000 |
| 55 | LNnDFH | Group 1 vs Group 2 | -4 | 0.3554 | 0.4738 |
| 56 | LNnDFH | Group 1 vs Group 3 | 5 | 0.3985 | 0.5198 |
| 57 | LNnDFH | Group 1 vs Group 4 | 6 | 0.4688 | 0.6049 |
| 58 | LNnDFH | Group 2 vs Group 3 | 9 | 0.1938 | 0.2736 |
| 59 | LNnDFH | Group 2 vs Group 4 | 10 | 0.2712 | 0.3774 |
| 60 | LNnDFH | Group 3 vs Group 4 | 1 | 0.9276 | 0.9714 |
| 61 | 3'SL | Group 1 vs Group 2 | 0 | 0.9835 | 0.9946 |
| 62 | 3'SL | Group 1 vs Group 3 | -1 | 0.9309 | 0.9714 |
| 63 | 3'SL | Group 1 vs Group 4 | -7 | 0.5183 | 0.6547 |
| 64 | 3'SL | Group 2 vs Group 3 | -1 | 0.9289 | 0.9714 |
| 65 | 3'SL | Group 2 vs Group 4 | -8 | 0.5394 | 0.6674 |
| 66 | 3'SL | Group 3 vs Group 4 | -7 | 0.6264 | 0.7442 |
| 67 | LNDFH-I | Group 1 vs Group 2 | 946 | 0.0000 | 0.0000 |
| 68 | LNDFH-I | Group 1 vs Group 3 | 898 | 0.0000 | 0.0000 |
| 69 | LNDFH-I | Group 1 vs Group 4 | 948 | 0.0000 | 0.0000 |
| 70 | LNDFH-I | Group 2 vs Group 3 | -48 | 0.5678 | 0.6900 |
| 71 | LNDFH-I | Group 2 vs Group 4 | 2 | 0.9880 | 0.9946 |
| 72 | LNDFH-I | Group 3 vs Group 4 | 50 | 0.6866 | 0.7922 |
| 73 | LNT | Group 1 vs Group 2 | -426 | 0.0000 | 0.0000 |
| 74 | LNT | Group 1 vs Group 3 | -198 | 0.0355 | 0.0576 |
| 75 | LNT | Group 1 vs Group 4 | -1352 | 0.0000 | 0.0000 |
| 76 | LNT | Group 2 vs Group 3 | 228 | 0.0314 | 0.0516 |
| 77 | LNT | Group 2 vs Group 4 | -926 | 0.0000 | 0.0000 |
| 78 | LNT | Group 3 vs Group 4 | -1154 | 0.0000 | 0.0000 |
| 79 | LDFT | Group 1 vs Group 2 | 337 | 0.0000 | 0.0000 |
| 80 | LDFT | Group 1 vs Group 3 | 233 | 0.0000 | 0.0000 |
| 81 | LDFT | Group 1 vs Group 4 | 337 | 0.0000 | 0.0000 |
| 82 | LDFT | Group 2 vs Group 3 | -104 | 0.0280 | 0.0467 |
| 83 | LDFT | Group 2 vs Group 4 | 0 | 0.9946 | 0.9946 |
| 84 | LDFT | Group 3 vs Group 4 | 105 | 0.1303 | 0.1955 |
| 85 | 6'-GL | Group 1 vs Group 2 | -9 | 0.0000 | 0.0001 |
| 86 | 6'-GL | Group 1 vs Group 3 | -9 | 0.0100 | 0.0175 |
| 87 | 6'-GL | Group 1 vs Group 4 | -5 | 0.2736 | 0.3774 |
| 88 | 6'-GL | Group 2 vs Group 3 | 1 | 0.8683 | 0.9387 |
| 89 | 6'-GL | Group 2 vs Group 4 | 4 | 0.3888 | 0.5127 |
| 90 | 6'-GL | Group 3 vs Group 4 | 4 | 0.5122 | 0.6538 |
| 91 | 3FL | Group 1 vs Group 2 | -1171 | 0.0000 | 0.0000 |
| 92 | 3FL | Group 1 vs Group 3 | 502 | 0.0000 | 0.0000 |
| 93 | 3FL | Group 1 vs Group 4 | 155 | 0.1805 | 0.2579 |
| 94 | 3FL | Group 2 vs Group 3 | 1673 | 0.0000 | 0.0000 |
| 95 | 3FL | Group 2 vs Group 4 | 1326 | 0.0000 | 0.0000 |
| 96 | 3FL | Group 3 vs Group 4 | -347 | 0.0123 | 0.0211 |
| 97 | LNFP-I | Group 1 vs Group 2 | 882 | 0.0000 | 0.0000 |
| 98 | LNFP-I | Group 1 vs Group 3 | -532 | 0.0000 | 0.0000 |
| 99 | LNFP-I | Group 1 vs Group 4 | 902 | 0.0000 | 0.0000 |
| 100 | LNFP-I | Group 2 vs Group 3 | -1414 | 0.0000 | 0.0000 |
| 101 | LNFP-I | Group 2 vs Group 4 | 20 | 0.9023 | 0.9667 |
| 102 | LNFP-I | Group 3 vs Group 4 | 1434 | 0.0000 | 0.0000 |
| 103 | LNFP-III | Group 1 vs Group 2 | -81 | 0.0000 | 0.0000 |
| 104 | LNFP-III | Group 1 vs Group 3 | 41 | 0.0387 | 0.0620 |
| 105 | LNFP-III | Group 1 vs Group 4 | -173 | 0.0000 | 0.0000 |
| 106 | LNFP-III | Group 2 vs Group 3 | 122 | 0.0000 | 0.0000 |
| 107 | LNFP-III | Group 2 vs Group 4 | -93 | 0.0015 | 0.0029 |
| 108 | LNFP-III | Group 3 vs Group 4 | -215 | 0.0000 | 0.0000 |
| 109 | LNnT | Group 1 vs Group 2 | 93 | 0.0000 | 0.0000 |
| 110 | LNnT | Group 1 vs Group 3 | 37 | 0.0214 | 0.0361 |
| 111 | LNnT | Group 1 vs Group 4 | 107 | 0.0000 | 0.0000 |
| 112 | LNnT | Group 2 vs Group 3 | -56 | 0.0017 | 0.0032 |
| 113 | LNnT | Group 2 vs Group 4 | 13 | 0.5692 | 0.6900 |
| 114 | LNnT | Group 3 vs Group 4 | 70 | 0.0080 | 0.0141 |
| 115 | LSTc | Group 1 vs Group 2 | 56 | 0.0000 | 0.0000 |
| 116 | LSTc | Group 1 vs Group 3 | -4 | 0.8022 | 0.8913 |
| 117 | LSTc | Group 1 vs Group 4 | 41 | 0.0728 | 0.1150 |
| 118 | LSTc | Group 2 vs Group 3 | -61 | 0.0014 | 0.0026 |
| 119 | LSTc | Group 2 vs Group 4 | -15 | 0.5395 | 0.6674 |
| 120 | LSTc | Group 3 vs Group 4 | 46 | 0.0990 | 0.1504 |
